# Tumor-Associated Macrophages: Protumoral Macrophages in Inflammatory Tumor Microenvironment

**DOI:** 10.34172/apb.2020.066

**Published:** 2020-08-09

**Authors:** Somaiyeh Malekghasemi, Jafar Majidi, Amir Baghbanzadeh, Jalal Abdolalizadeh, Behzad Baradaran, Leili Aghebati-Maleki

**Affiliations:** ^1^Department of Basic Oncology, Oncology Institute, Hacettepe University, Sihhiye, Ankara, TR-06100, Turkey.; ^2^Immunology Research Center, Tabriz University of Medical Sciences, Tabriz, Iran.; ^3^Department of Immunology, School of Medicine, Tabriz University of Medical Sciences, Tabriz, Iran.; ^4^Drug Applied Research Center, Tabriz University of Medical Sciences, Tabriz, Iran.

**Keywords:** Tumor-associated macrophage (TAMs), Tumor microenvironment (TME), Therapeutic target, Malignant cells

## Abstract

Tumor microenvironment consists of malignant and non-malignant cells. The interaction of these dynamic and different cells is responsible for tumor progression at different levels. The non-malignant cells in TME contain cells such as tumor-associated macrophages (TAMs), cancer associated fibroblasts, pericytes, adipocytes, T cells, B cells, myeloid-derived suppressor cells (MDSCs), tumor-associated neutrophils (TANs), dendritic cells (DCs) and Vascular endothelial cells. TAMs are abundant in most human and murine cancers and their presence are associated with poor prognosis. The major event in tumor microenvironment is macrophage polarization into tumor-suppressive M1 or tumor-promoting M2 types. Although much evidence suggests that TAMS are primarily M2-like macrophages, the mechanism responsible for polarization into M1 and M2 macrophages remain unclear. TAM contributes cancer cell motility, invasion, metastases and angiogenesis. The relationship between TAM and tumor cells lead to used them as a diagnostic marker, therapeutic target and prognosis of cancer. This review presents the origin, polarization, role of TAMs in inflammation, metastasis, immune evasion and angiogenesis as well as they can be used as therapeutic target in variety of cancer cells. It is obvious that additional substantial and preclinical research is needed to support the effectiveness and applicability of this new and promising strategy for cancer treatment.

## Introduction

The relationship between cancer and inflammation were observed in the nineteenth century and the most of tumors often occurred in chronic inflammatory sites.^1^ In fact, inflammatory microenvironment has been identified as an integral component of carcinogenesis.^2^ The hallmark of tumor-promoting inflammation include the presence of inflammatory cells and inflammatory mediators in the tumor stromal cells. The formation of inflammatory microenvironment mediates by genetic events and immune cells such as regulatory T cells, myeloid-derived suppressor cells (MDSCs), tumor-associated neutrophils (TANs), regulatory B cells and tumor-associated macrophages (TAMs). These heterogeneous cells interact with tumor cells to contribute the tumor initiation, promotion and metastases.^3^ TAMs are the major component of tumor-associated stromal cells that orchestrated cancer-related inflammation.^4^ The main features of macrophages are heterogeneity and plasticity in tumor microenvironment and TAMs have either tumor-promoting or prevention role upon different stimuli. Macrophages can be polarized into different phenotypes: classically activated macrophages M1 and alternatively activated macrophages M2 in tumors. M1-like macrophages activated by interferon-γ (IFN-γ) and lipopolysaccharide (LPS) which produced pro-inflammatory cytokines like IL-12, IL-23, TNF-α and IL-6 that promote Th1 responses. In contrast, IL-4, IL-10, IL-13 and TGF-β stimulated M2-like macrophages which induce Th2 responses.^5^ TAMs enhance tumor angiogenesis, metastases, tissue repair, extracellular matrix (ECM) degradation and suppress immune responses.^6^ However, the extremely complicated relationship between TAMs and malignant tumor cells remains a subject of controversy. On the other hand, the multifaceted role of TAMs in tumor progression, they are now being as a therapeutic target, diagnostic and prognosis markers for cancer. In this review, we discuss how TAMs mediated tumor progression and we summarized novel molecule and mechanism involved in macrophage polarization and recruitment offers novel therapeutic approaches.

## Origins of TAMs

**Figure 1:**
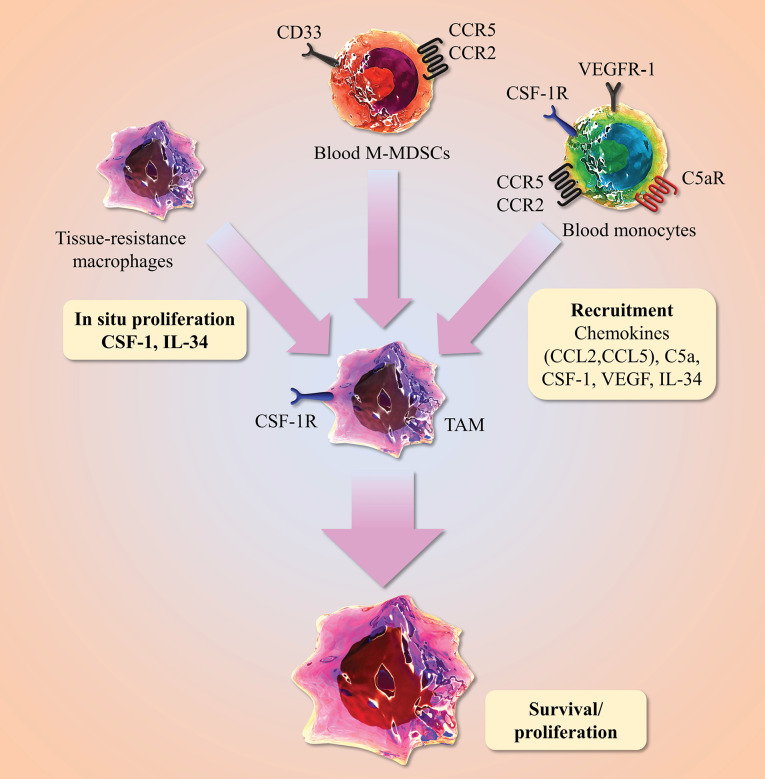
**Schematic representation of the origin and polarization of TAMs in tumor microenvironments. ** Blood monocytes, tissue resident macrophages and monocytic myeloid-derived suppressor cells (M-MDSCs) can be recruited to tumour stroma in response to diverse chemokines and cytokines. Blood monocytes and M-MDSCs are recruited and polarized into macrophages in response to various chemokines and cytokines including, CSF-1, VEGF, IL-34, CCL2, CCL5and complement components (C5a) which produced by stromal and tumor tissues. Local tissue-resident macrophages and tumour-infiltrating monocytes differentiate into TAMs. Moreover, distinct populations of TAMs in some tumours can be proliferated.

The first line of defense mediated by innate immune response like macrophages, which participate in immune responses, tissue repair and homeostasis.^7^ Recent studies in pancreatic cancer shows skepticism about the origin of TAMs from hematopoietic stem cells and they proved that TAMs derived from embryonic precursors or primitive yolk sac precursors referred to tissue-resident macrophages with self-renewal capability.^8^ Movahedi and colleagues described that which two main circulating monocytes, “Ly6C^+^ inflammatory” or “Ly6C- resident” monocytes, is the major source of TAM in mice.^9^ They injected labeled Ly6C^hi^ and Ly6C^lo^ monocytes into tumor-bearing mice. They found that inflammatory monocytes contain TAM precursor cells.^10^ A comparative gene expression profiling from murine tumor microenvironment revealed that TIE-2 expression monocytes (TEM) and TAM profiles were related each other. Resident macrophages and TIE-2 embryonic macrophages express a gene signature closely related to circulating TEM. On the other hand, TAMs express a gene profiling more related to inflammatory macrophages and compared to TAM, TEM show enhanced angiogenic activity with lower pro-inflammatory activity. However, the relationship between TAM and TEM are elusive and additional studies are needed.^11^ Metastatic breast cancer patients have elevated level of TEM which monocytes express (CD11b^+^, CD14^+^CD45^+^ cells, TIE-2) in their peripheral blood and in the breast tumor microenvironment.^12^ In gliomas, TAMs derived from resident microglial cells of embryonic origin, infiltrated blood monocytes and monocytic M-MDSCs.^13^ STAT3 is a key transcription factor induce the polarization of M-MDSCs into mature TAMs.^14^ The polarization of mouse inflammatory monocytes (Ly6C^+^/CCR2^+^cells) into TAMs mediated by a major transcriptional effector of Notch signaling like RBPJ and down regulation of this protein in TAMs reduced the tumor size in mouse breast cancer.^15^ Chemoattractants, cytokines such as CSF-1, VEGF, and IL-34, chemokines like CCL2 and CCL5, and complement components (C5a) responsible for recruitment of inflammatory monocytes and monocytic myeloid-derived suppressor cells (M-MDSCs) into the tumor microenvironment.^16^ Indeed, such chemotactic factors activate transcriptional factors that contributes the differentiation of macrophage. The binding of CCL18 to its receptor PITPNM3 recruits macrophages in human breast cancer model with the collaboration of CSF2 mediators.^17^ An important player in the recruitment of monocytes to the tumor, is considered CCL2-CCR2 axis that has been proposed as a new therapeutic target.^18^ There is controversial debate about the exact origin of CCL2 within tumor. Zhou et al found that TANs as a main source of CCL2 and CCL17 in HCC, which adsorbed the macrophages and CCR4^+^ Treg cells to the tumor tissue.^19^ In return, Spary et al identified CCL2 derived fibroblasts recruited the monocytes in prostate cancer.^20^ On the other hand, CCL2 induce the production of CCL3 in human and murine macrophages, which this CCL3-CCR1 axis promotes metastasis in the mouse model^21^ (Figure 1).

## Polarization of TAMs

Based on their presence in the tumor microenvironment, TAMs associated with phenotypic plasticity, intratumor and intertumor diversity, ultimately, they polarized toward immunosuppressive phenotype. TAMs characterized as M2-like macrophage, which express surface molecules include CD204 ( macrophage scavenger recep­tor A ), CD163, CD206 (MRC1), CD301, stabilin-1 (scavenger receptor and adhesion molecule), dectin-1, DC-SIGN (dendritic cell-specific intercellular adhesion molecule-3-grabbing non-integrin), chemokine (such as CCL17, CCL18, CCL22 ), cytokine (such as IL-10, IL-1ra, decoy IL-1RII), vascular endothelial growth factor (VEGF), arginase I, Fizz1 (resistin-like beta, also known as Fizz1) and Ym1 (chitinase 3-like 3, also known as Ym1). M2-like macrophage polarization induce by IL-4, IL-13, Toll-like receptor and IL-10.^22,23^ M1-like macrophages polarized with (for example, LPS, IFN-γ and TNF-α), usually express high level of HLA-DR, iNOS which produced proinflammatory cytokines like TNF-α, IL-1β, IL-6 and IL-12.^24^ TAMs by bidirectional interaction can promote immunosuppressive of regulatory T cells.^25^ TAMs facilitated tumor proliferation and metastasis by production of MMPs, cathepsins, FGF, VEGF, PDGF and various chemokines like CXCL8.^26^ CSF-1, is a monocyte attractant in TME, which promote tumorigenesis that derive macrophages polarization toward M2-like phenotypes. In contrast, GM-CSF activate antitumor activity of macrophages.^27^ In pancreatic ductal adenocarcinoma, cancer-associated fibroblasts in TME produced GM-CSF and induce M2 polarization.^28^ A recent study showed that the anti-inflammatory lectin REG3β enhanced the polarization of M2-like phenotype in an orthotopic pancreatic cancer of mouse model.^29^ REG4 another lectin, involved in macrophage polarization toward M2 phenotype in pancreatic cancer by induction of EGFR/AKT/CREB signalling pathway.^30^ Exosomal miR-301a-3p maintain M2 phenotype during hypoxic condition via activation of PTEN/PI3Kγ signaling pathway in pancreatic cancer metastasis.^31^ Biglycan and hyaluronan, Tumor-derived ECM components, induced TAM polarization via TLR2 and TLR4.^32^ Recently, micro-RNAs as regulator of gene expression could be used as a biomarkers in pathogenesis of cancer and inflammatory diseases. M1 macrophages express miR-125, miR-155 and miR-378 and M2 macrophages upregulated miR-9, miR-21, miR-146, miR-147, miR-187 and miR-511-3p.^33^ In particular, miR-155 induce the polarization of macrophages toward M1 phenotype by regulation of NF-Kβ signaling pathway in response to LPS and IFN-γ.^34^ Bouhlel et al found that PPAR-*γ* (peroxisome proliferator-activated receptor gamma) as a type-II nuclear receptor differentiated monocytes toward M2-like macrophages. PPAR-*γ* express in adipose tissue, colon and macrophages and regulate fatty acid storage and glucose metabolism.^35^ TLR4 agonist like heat-treated *Mycobacterium indicus pranii (Mw)* in combination with DTA-1(an agonistic antibody for glucocorticoid-induced TNFR-related protein (GITR)) induce the repolarization of TAMs into M1-like macrophages by a significant increase in IL-12, iNOS and HLA-DR in a mouse model of advanced stage melanoma.^36^

## TAM functions in tumor microenvironment

### TAM in inflammation

**Figure 2:**
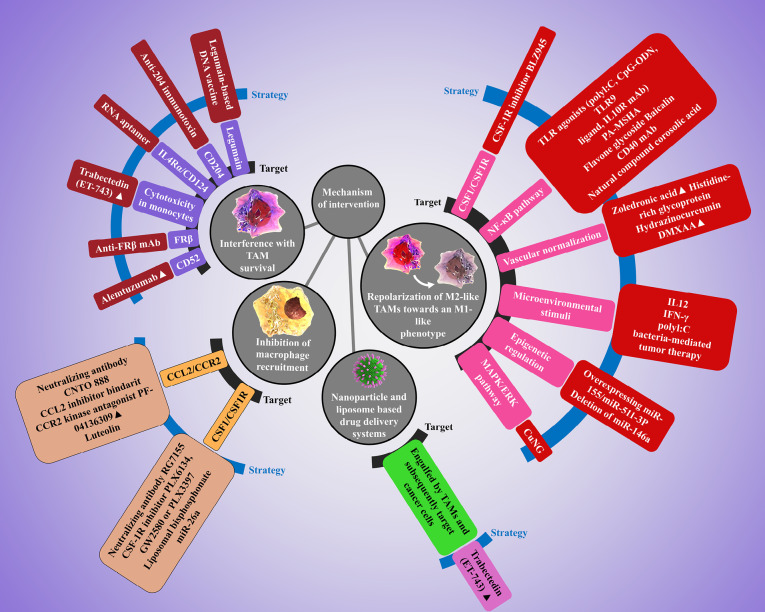
**The effect of TAMs on tumor promotion.** The protumor function of TAMs including: angiogenesis, metastasis and invasion, epithelial-to-mesenchymal transition, proliferation, immune evasion and inflammation.

The relationship between inflammation and cancer can be classify into two pathways: the intrinsic pathway and the extrinsic pathway. The intrinsic pathway driven by genetic events include the activation of proto-oncogene by mutation, inactivation of tumor-suppressor gene, chromosomal amplification and deletion. The extrinsic pathway is activated by inflammatory conditions at certain anatomical sites (such as colon, pancreas, prostate). The converging of two pathways resulting in the activation of NF-KB transcription factor, HIF-1α) and signal transducer and activator of transcription 3 (STAT3) in tumor cells. These transcription factors induce the production of inflammatory cytokines, chemokines as well as prostaglandins. These mediators recruited leukocytes, mostly monocytes, resulting in the production of cancer-related inflammatory microenvironment.^37^ TAMs are an important leukocyte infiltration in TME that connected inflammation and cancer.^38^ TAMs in mouse and human tumors have M2 phenotype, which promote tumor progression, angiogenesis, remodeling tissues, metastasis and suppression of adaptive immunity. Signals derived from regulatory T cells and tumor cells like IL-10, TGF-β and M-CSF differentiated M2 phenotype in tumor tissue.^39^ Cancer related inflammation have dual potential features and may be affected by tissue type. Psoriasis is a chronic inflammatory disease that is not related to an increased risk of skin cancer because it is a T helper1-cell-mediated disease. In some tumor subtypes like eosinophils in colon tumors, TAMs in a subset of breast tumors and pancreatic tumors, the presence of inflammatory cells associated with better prognosis.^40^ Evidences showed that NF-KB determine protumour and anti-tumour responses in macrophages.^41^ More recently, patients with bladder cancer treated by administering *Mycobacterium bovis* bacillus Calmette–Guerin. This treatment induced the polarization macrophages toward M1 phenotype by triggering of TLR receptors.^42^ Multiple evidence indicated that immune inflammatory cells in neoplasia can be promote tumor progression, angiogenesis and invasion. Necrotic cells can release pro-inflammatory factors, such as IL-1α into tumor microenvironment.^5^ TAMs promote the survival of inflammatory breast cancer IBC by expression of gene encoding the AXL/GAS6 (growth arrest- specific protein 6) signaling.^43^ Versican, an extracellular proteoglycan, which activate macrophages via TLR2 and TLR6 in lung cancer. TLR2/6 enhanced LLC metastasis growth by secretion of TNF-α from myeloid cells^44^ (Figure 2).

### TAM in angiogenesis

Like healthy tissues, tumors need to create a bloodstream to supply their oxygen and nutrients and other metabolic functions.^45^ This achieved through angiogenesis, which consists of formation a new blood vessel from circulating endothelial progenitor cells and pre-existing vessels.^46^ HIF is an important signals regulating angiogenesis process because they transcribed the genes responsible for inducing angiogenesis like vascular endothelial growth factor (VEGF-A). The pro-angiogenesis capacity of TAMs depended on secretion of growth factors and inflammatory cytokines by promoting EC survival, proliferation and activation.^47^ TAMs are a major source of VEGF-A in mice and human. The elimination of VEGF-A in TAMs hindrance angiogenic switch and weaken the formation of tumor-associated blood vessels in mouse cancer models.^48^ Another pro-angiogenic factors secreted by TAMs include placental growth factor (PIGF), VEGF-C, IL-1β, IL-6, TNF, CXCL8 (IL-8) and fibroblast growth factor 2.^49^ TAMs express WNT signaling pathway and the deletion of WNT7b in TAMs decreased the vascular density in mouse mammary carcinomas.^50^ TAMs secret soluble and membrane-bound proteases include MMP2, MMP9, MMP12 and cathepsin that degrade ECM to release the sequestered pro-angiogenic factors.^51^ Accordingly, TAMs that express ANGPT receptor TIE2 (also known as TEK) increased the vascular density and metastasis in some tumors.^52^ Hypoxia induce the expression of CXCL12 and ANGPT2 in tumor tissue, which recruited the CXCR4 ^+^TIE2 ^+^ TAMs.^53^ Genetic deletion of TIE2 block ANGPT2-TIE2 signaling pathway in TAMs, result in decrease angiogenic interaction.^54^ Notch signaling in TAMs associated with pathological angiogenesis but the role of this pathway in tumor angiogenesis were not elucidated.^55^ TAMs express semaphorins, vascular guidance molecules, which mediate EC survival and migration.^56^ TAMs by induction of IL-10 and STAT3/Bcl-2 signaling pathway are able to inhibit breast cancer apoptosis upon paclitaxel treatment.^57^ Wenes and colleague reported that TAM metabolism and REDD1 are as the first potential target for blood vessel formation. Hypoxia induce the expression of regulated in development and DNA damage response 1 (REDD1), which orchestrate the tumor angiogenesis.^58^ Toge et al investigated that TAM counts were increased in renal-cell carcinoma, showing the elevated level of TAM counts and VEGF among angiogenic factors like PyNPase (pyrimidine nucleoside phosphorylase), MVD (factor VIII), CD34 and pTstage.^59^

### TAM in metastasis

The great majority of cancers arise from epithelial cells, yielding carcinomas. In order to carcinomas cells acquire motility and invasiveness, they undergo alteration of the epithelial–mesenchymal transition. The hallmark of epithelial cells, E-cadherin and cytokeratins, is repressed, while the component of mesenchymal cells, vimentin and N-cadherin, is induced. In general, studies shown that the interaction between TAMs and malignant cell are required for invasion and metastasis. The movement of cancer cells depend on secretion of EGF from TAM and production of CSF-1 from tumor cells.^60^ In breast cancer, CSF-1 secreted from tumor cells recruit monocytes from circulation and these cells differentiated into TAMs, its in turn produced the EGF.^61^ Local secretion of EGF stimulate EGF receptor on breast cancer cells, which induce the SOX-2 gene through activation of STAT3 signaling pathway.^62^ The expression Wiskott–Aldrich syndrome protein in TAMs induce mammary carcinoma metastasis and invasion by induction of EGF production from macrophages and migration of macrophages toward CSF-1 from cancer cells.^63^ Finally, cancer cell derived GM-CSF induce the secretion of CCL18 from mammary TAMs, which trigger integrin clustering in cancer cells and mesenchymal-like phenotype via activation of NF-KB that mediate adherence to the ECM.^64^ Metastasis required dissemination of cells from primary tumor, intravasate into lymphatic and blood microvessels, extravasate at distant sites. In breast cancer, invasive isoform, MENA^INV^ cancer cells and TAMs migrate toward blood vessels by EGF-CSF1 paracrine loop. Mena-overexpressing tumor cell, proangiogenic TIE2^Hi^/VEGF^Hi^ macrophage and the endothelial cell make TMEM (tumor microenvironment of metastasis).^65^ A unique population of monocytes in pritumoural stroma of HCC express c-Met molecule, which associated with poor survival of patients. These monocytes produced MMP-9 in response to the HGF derived tumor stromal.^66^ Macrophages that support metastatic of cancer cells express surface markers like VEGFR1, CCR2, and CX3CR1, which different from angiogenic macrophages express molecules (such as TIE2 or CXCR4).^11^ Recent studies demonstrated that CCR2 trigger the production of CCL3 from macrophages in breast cancer mouse model. CCL3 via CCR1 signaling promote metastasis in lung and breast cancer ^21^. Recent studies indicated that hypoxic mammary tumors secret lysyl oxidase (LOX) to recruit CD11b+ myeloid cell at metastasis sites and these cells produce MMP-2 to disport collagene IV in experimental breast cancer metastasis models. Additionally, the elimination of LOX prevent metastasis burden into pulmonary.^67^

### TAM in immune evasion

Immune system plays an important role in eradicating formation of incipient neoplasias and micrometastasis, but solid tumors managed to avoid detection. TAMs derived CCL17, CCL18 and CCL22 adsorbed Treg to tumor stroma, which result in the promoting of immunosuppressive activity of regulatory T cells by immunosuppressive cytokines, including IL-10 and TGFβ.^68^ Indoleamine 2, 3-dioxygenase in the tumor microenvironment and TAM breakdown tryptophan, which result in the suppression of T cell and dendritic cell activity.^27^ Prostaglandins like COX-1 and COX-2 in TAMs have immunosuppressive effects on T cells.^69^ PD-L1 and PD-L2 to be expressed in TAMs and tumor cells, which promote the inhibitory function of PD-1 immune checkpoint, B7-H4 and VISTA in T cells.^70^ Oncogenic MYC in cancer cells induce the expression of CD47, and the immune-checkpoint protein PD-L1. CD47 perform as a ‘don’t eat me’ signal that suppress innate immunity through phagocytic activity of macrophage and PD-L1 inhibit adaptive immune responses.^71^ In pancreatic cancer, infiltrating Treg in tumor microenvironment upregulated CTLA-4 (cytotoxic T-lymphocyte antigen 4) and PD-1, thus, blockage of these pathways enhance anti-tumor immunity.^72^ TAMs with CD120a, CD120b secret NO, resulting in promoting the apoptosis of activated T cells in tumor tissue.^73^ In pancreatic cancer, CD11b^+^myeloid cells inhibit CD8^+^Tcells by induction of PD-L1 in tumor cells in an epidermal growth factor receptor (EGFR)/mitogen-activated protein kinases-dependent manner.^74^ TAM derived TGF-β reduced dendritic cells migration, antigen presentation and adaptive immune responses. Recent studies indicated that TAMs trigger CD27^low^CD11b^high^-exhausted NK-cell phenotype and inhibit cytolytic activity of NK cells by TGF-β depended manner.^75^ Additionally, hypoxia promote TAM derived CCL20 via activation of NF-KB signaling pathway. CCL20 induce the recruitment of Vα24-invariant NKT cells to the hypoxic TME, where the antitumor function and viability of NKT cells were repressed.^76^ TAMs produce CCL2 which induce CCR2^+^ monocytic MDSCs migration from bone marrow to tumor. However, tumor infiltrating MDSCs polarized toward TAM by CSF-1 and HIF-1α. In human glioblastoma, elevated level of CCL2 correlated with increased counts of TAMs and reduced survival of patient.^77^

### TAM in therapeutic target in cancer

**Figure 3:**
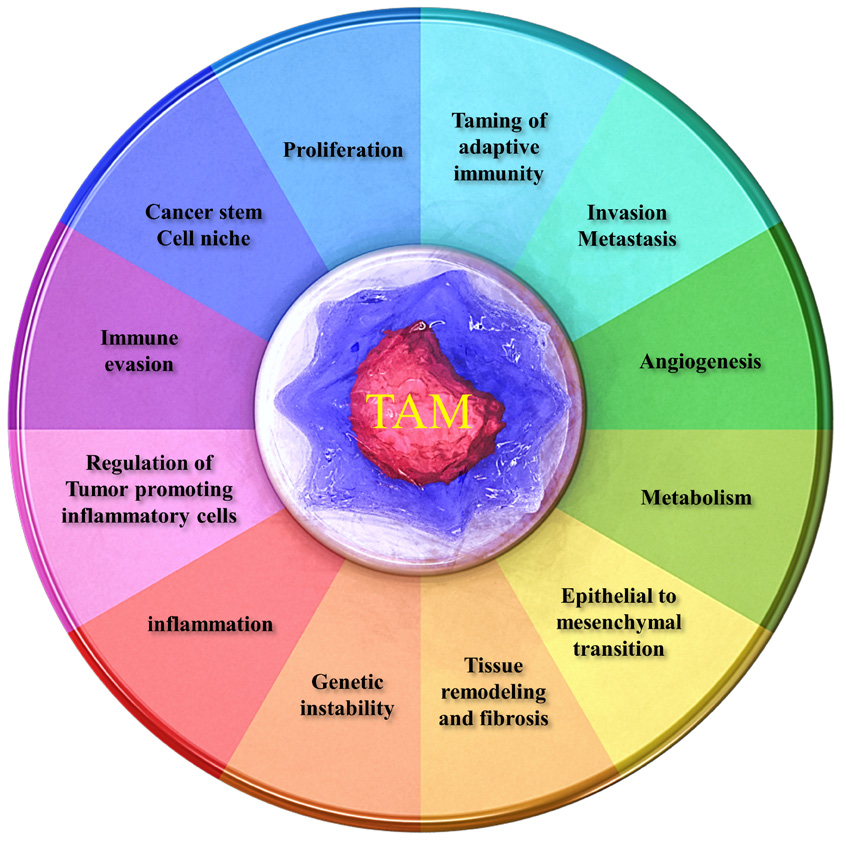
**A schematic representation of the clinical approaches of agents that target TAMs.** These immunotherapeutic strategies include interference with TAM survival, limiting of macrophage recruitment, targeting TAMs with radiotherapy and reprogramming of tumor-promoting M2- like TAMs to antitumor macrophages.

TAM- targeting immunotherapy represent an effective strategy for cancer treatment. These immunotherapeutic strategies include interference with TAM survival, limiting of macrophage recruitment, targeting TAMs with radiotherapy and reprogramming of tumor-promoting M2- like TAMs to antitumor macrophages (Figure 3).

## Interference with TAM survival

Trabectedin (ET-743) induce apoptosis in monocytes. This function mediated by activation of caspase-8, which plays pivotal role in the extrinsic apoptotic signaling pathway via Fas and TNF-related apoptosis inducing ligand receptors.^78^ Liposome-encapsulated bisphosphonate clodronate can be phagocytized by macrophages leads to macrophages deletion and inhibit tumor progression. In contrast, liposomal trabectedin induce the apoptosis of all macrophages by activation of caspase-8.^79^ M2pep target specially with high affinity for M2-like macrophages in murine and subsequently improve the survival of tumor-bearing mouse.^80^ Legumain express in TAM in murine breast cancer tissue. A legumain-based DNA vaccine promote the activation of CD8^+^Tcells and override M2-like macrophage in the metastasis of breast, colon and lung in mice.^81^ An RNA aptamer activate CD8^+^Tcells by targeting murine or human IL4Rα/CD124 on TAMs.^82^ IL-27 induce the apoptosis of M2-like macrophages and proliferation, invasion of pancreatic cells. It is also as a novel therapy when combine with gemcitabine and both of them target TAMs in pancreatic cancer.^83^

## Limiting of macrophage recruitment

CCL2 synthesis by tumor cells, stromal and bone marrow osteoblasts, subsequently mediate tumorigenesis, metastasis and recruitment of inflammatory monocytes that express CCL2 receptor CCR2 to the tumor sites. Blockage of CCL2 and CCR2 suppresses M2 macrophage migration. A CCL2 blockage agent (anti-human CNTO888, carlumab and anti-mouse C1142) in combination with docetaxel induce tumor regression in prostate cancer. A CCR2 kinase antagonist PF-04136309 inhibit M2 macrophage migration in murine pancreatic cancer.^84^ CSF1-CSF1R regulate macrophage recruitment, proliferation and differentiation. A CSF1R inhibitors PLX6134, GW2580 and PLX3397 inhibit TAM infiltration by induction of CD8^+^Tcells activity. Additionally, the monoclonal antibody (mAb) RG7155 against CSF1R reduce TAM recruitment.^85^ The CSF1R inhibitor BLZ945 reduced M2-associated genes such as arginase 1 and CD206 in a mouse proneural glioblastoma model. BLZ945 inhibit the activity and proliferation TAMs and reprograme tumor promoting TAMs to antitumor macrophages.^86^

## Reprogramming of M2- like TAMs to M1-like phenotype

Repolarization of M2-like macrophages to anti-tumor phenotype under physiological conditions is a crucial strategy for cancer therapy. In Lewis lung carcinoma mice model tumor, introduction of polyinosinic:polycytidylic acid (polyI:C) into mice activate the TLR3/Toll–IL1 receptor, which reprogrammed M2-like macrophage toward tumor suppressor phenotypes.^87^ Injection of attenuated *Listeria monocytogenes* into the tumor stroma of ovarian cancer mice model induce tumor cell lysis through synthesis of nitric oxide, resulting in the switch of TAM toward anti-tumor phenotypes.^88^ Similarly, in B16F10 melanoma tumors, introduces of heat-killed *Mycobacterium indicus pranii* in TME induce the repolarization of M2-like phenotype into M1 macrophages.^36^ In spontaneous mammary carcinogenesis, the anti-angiogenic agent like zoledronic acid induce reprogramming of pro-angiogenic TAMs toward tumor suppressor phenotype by vascular normalization.^89^ The STAT3 phosphorylation inhibitor such as hydrazinocurcumin switch M2-like phenotype to an M1-like phenotype to inhibit angiogenesis and metastasis in breast cancer.^90^ In mouse models of non-small-cell lung cancer, 5, 6-dimethylxanthenone-4-acetic acid switch TAM into M1-like phenotype by promoting the vascular disrupting via STING

Activation.^91^ In gastric carcinoma progression in mice, *Pseudomonas aeruginosa* strain (PA-MSHA) induce the switch of M2-like phenotype toward M1 macrophages upon activation of NF-KB signaling pathway.^92^ Introduction of anti-CD40 in spontaneously develop pancreatic ductal adenocarcinoma in KPC mice promote the polarization of TAMs toward M1-phenotypes upon activation of IL12, TNFα and INF-γ.^93^

## Targeting of TAM with radiotherapy

Radiotherapy is used for treatment of more than 50% cancer patient and associated with tumor regression in majority of cancers. However, macrophages have radioresistance property because of having a manganese superoxide dismutase, ROS, RNS and a scavenger of superoxide ions. On the other hand, RT can induce the recruitment of macrophages into the tumor tissue by stimulation of CCL2, CSF1 production and promote the tumor progression. Depletion of macrophages by liposomal clodronate before IR and the inhibition of CSF1 receptor with PLX3397 can promote the anti-tumor effect of RT.^94^ Crittenden and his colleagues reported that high doses of irradiation produce M2 phenotype through p50–p50 NFκB homodimer activation and IL-10 production. Similarly, low doses of irradiation reduce the translocation of p50–p65 NFκB into nucleus in M1 macrophages. Moderate doses of irradiation shift macrophages toward M1 phenotype by the activation of NF-κB p65.^95^

## Conclusion

Monocyte-macrophage lineage an important component of TME has been identified as a crucial factor in the proliferation and cancer cell progression. Moncytes can be recruited into tumor sites by chemotactic factors, which produce from tumor cells and tumor stroma. Monocytes transformed into M2-like TAM to facilitate tumor angiogenesis, invasion and metastasis. These distinctive phenotype derived from the plasticity nature of macrophages in the tumor microenvironment. TAMs to apply clinically, as a diagnosis and prognosis marker and a therapeutic target as well. Anti-tumor therapeutic strategy include reducing TAM survival, limiting macrophage recruitment and skewing M2-like TAMs into an M1-like phenotype. Among these strategies, switching TAM toward M1 macrophages is most promising because these therapies do not destroy macrophages also can be useful method for tumoricidal activity in the tumor tissue to reduce tumor progression. Recent study identify that TLR ligands promote repolarization of macrophage in mouse models.^96^ Additionally, combination of TAM repolarization with checkpoint inhibitors (PD1 or CTLA4 antagonists), stimulating antibodies (CD40 or GITR agonists) and radiotherapy displayed a remarkable strategies for cancer treatment.^97^ Vessel normalization susceptible tumor cells to chemotherapy agents. Anti-VEGF and anti-angiopoietin treatment leading to vessel normalization, reduce hypoxia, induce TAM repolarization and improve CTL, NK cell infiltration.^98^ Several drugs are used clinically targeting TAM such as trabectedin reduce TAM survival and alemtuzumab eliminates TAMs by targeting a TAM surface protein.^99^ Therefore, more investigation are required to assess the elevated ratio of M1-like macrophage to M2-like macrophage to identify tumor prognosis and prevent tumor progression. In addition, TAM population associated with poor prognosis in patient, well-define criteria are essential to evaluated macrophage population.

## Ethical Issues

Not applicable.

## Conflict of Interest

Authors declare no conflict of interest.

## Acknowledgments

This work was supported financially by Immunology Research Center, Tabriz University of Medical Sciences, Tabriz, Iran.
